# LncRNA Taurine Upregulated Gene 1 as a Potential Biomarker in the Clinicopathology and Prognosis of Multiple Malignant Tumors: A Meta-Analysis

**DOI:** 10.1155/2021/8818363

**Published:** 2021-03-02

**Authors:** Qi Huang, Jingjing Wu, Hui Wang, Na Li, Zhen Yang, Mingjun Zhang

**Affiliations:** ^1^Department of Oncology, The Second Affiliated Hospital of Anhui Medical University, Hefei, Anhui, China; ^2^Cancer Institute (Key Laboratory of Cancer Prevention & Intervention, National Ministry of Education, Provincial Key Laboratory of Molecular Biology in Medical Sciences), Second Affiliated Hospital, Zhejiang University School of Medicine, Hangzhou, China

## Abstract

**Background:**

The lncRNA taurine upregulated gene 1 (TUG1) is a recently identified potential biomarker in cancer. However, its prognostic role in various cancers is inconsistent among published data. We conducted this meta-analysis to comprehensively confirm the prognostic effect of TUG1 in malignant tumors.

**Methods:**

We systemically analyzed the prognostic-predictive capacity of TUG1 through amplifying sample sizes and cancer types. STATA 12.0 was applied for this meta-analysis.

**Results:**

A total of 57 eligible studies were included in our meta-analysis. The pooled results suggested that overexpression of TUG1 was significantly correlated with unfavorable overall survival (OS) (HR = 1.70, *p* < 0.001), shorter recurrence-free survival (RFS) (HR = 2.40, *p* ≤ 0.001), and shorter event-free survival (EFS) (HR = 1.88, *p* < 0.001) in patients with cancer. In the subgroup analysis by cancer type, elevated TUG1 expression was associated with poorer survival in patients with gastrointestinal cancer, urinary tumors, gynecological tumors, hematological tumors, and osteosarcoma. However, high expression of TUG1 in respiratory tumors indicated a better prognosis. There was no correlation between high TUG1 expression and OS in patients with head and neck neoplasms or melanoma. Additionally, overexpression of TUG1 was found to be correlated with low-grade tumor differentiation, advanced tumor stage, positive lymphatic metastasis, and positive distant metastasis.

**Conclusions:**

High TUG1 expression correlates with poor prognosis and advanced clinicopathological features, verifying the prognostic-predictive capacity of TUG1 in tumors, especially in gastrointestinal cancer, urinary tumors, gynecological tumors, hematological tumors, and osteosarcoma. Meanwhile, the prognostic role of TUG1 in respiratory tumor may be opposite to other tumors.

## 1. Introduction

Long noncoding RNAs (lncRNAs) are an emerging class of vital regulators participating in various biological functions and disease processes to different degrees [1, 2]. Next-generation sequencing has revealed that specific lncRNAs are mutated or aberrantly expressed in cancers, and the specific role of lncRNAs in different tumors is yet to be annotated [3].

The lncRNA taurine upregulated gene 1 (*TUG1*) has been reported to exert oncogenic or tumor suppressive function in cancer through altering cancer-related gene expression at the transcriptional level. According to previous studies, TUG1 was found to be upregulated and oncogenic in a broad spectrum of cancers, including colorectal cancer, bladder cancer, esophageal squamous cell carcinoma, and osteosarcoma [4–7]. Meanwhile, in some types of breast cancer, non-small-cell lung cancer, and glioma, TUG1 was expressed at a low level when compared with noncancerous tissues and acts as a tumor suppressor [8–10]. Meta-analyses have attempted to demonstrate the potential diagnostic or prognostic role of TUG1 in cancer, but the conclusions were not consistent [11–18]. In recent years, studies examining TUG1 expression have been conducted through high-throughput whole-genome sequencing or quantitative real-time polymerase chain reaction (qRT-PCR), and we carried out the current meta-analysis to evaluate the role of TUG1 in tumors by expanding the number of samples and tumor types. This research may provide additional evidence for TUG1 in predicting the prognosis of tumors.

## 2. Materials and Methods

### 2.1. Literature Search Strategy

Until January 15, 2021, relevant literature concerning the expression of the lncRNA TUG1 in cancer was extracted from databases including PubMed, Embase, and Web of Science, together with three Chinese databases: China National Knowledge Infrastructure (CNKI), Wanfang, and Weipu. Key terms and all possible combinations were as follows: ‘taurine upregulated gene 1 OR TUG1' AND ‘cancer OR tumor OR neoplasm OR carcinoma.' The reference lists of all primary studies were also examined to identify additional eligible studies.

### 2.2. Study Selection

All eligible literature included in our meta-analysis met the following criteria: (1) The expression of the lncRNA TUG1 was measured in human tumors, and patients were grouped according to the expression levels of TUG1. (2) Assessment of the relationship between TUG1 expression and overall survival (OS), progression-free survival (PFS), disease-free survival (DFS), recurrent-free survival (RFS), event-free survival (EFS), or clinical-pathological parameters such as tumor differentiation, tumor stage, and metastasis. (3) Sufficient information was provided to estimate the hazard ratio (HR) or odds ratio (OR) and their 95% confidence intervals (CIs).

The articles were excluded if they had the following characteristics: (1) letters, reviews, case reports, and conference abstracts without original data; (2) laboratory studies conducted at the cellular level only; (3) lack of available data or survival curves to compute HRs, ORs, or the corresponding 95% CIs, and (4) multiple duplicate publications with overlapping populations, excluding the smaller sample cohort.

### 2.3. Data Extraction and Assessment of Study Quality

Two investigators (Jingjing Wu and Hui Wang) independently extracted data and assessed study quality using a standardized form. Any discrepancy was arbitrated by a third reviewer (Qi Huang). The following characteristics were retrieved: first author's name, publication year, country of patients' origin, tumor type, sample size, number of patients in the TUG1 level group, tumor stage, detection method of TUG1 expression, survival data (obtained directly or extracted from Kaplan-Meier survival curve), clinical-pathological data, and Newcastle-Ottawa Quality Assessment Scale (NOS) score.

The quality assessment of eligible studies was in accordance with the NOS. Our quality score was judged on three sections: selection, comparability, and exposure or outcome. With a mean score of 6.9 from enrolled studies, we defined studies scored 7 or above as high quality.

### 2.4. Statistical Analysis

All data analysis was performed using STATA software version 12.0 (Stata Corporation, College Station, TX, USA). We calculated the pooled HRs and the 95% CIs of the included articles to assess the impact of TUG1 on patient prognosis and clinical-pathological characteristics. OS, PFS, DFS, RFS, and EFS were all included in outcome analyses. HRs and their corresponding 95% CIs described in the literature were adopted directly. Otherwise, they were extracted from Kaplan-Meier curves by Engauge Digitizer version 4.1 (http://digitizer.sourceforge.net/). Additionally, we computed the ORs and their 95% CIs to explore the correlation between TUG1 expression and the clinical-pathological parameters of all tumors. In our analysis, an HR > 1 indicated that a high expression of TUG1 was an unfavorable factor in cancer, and an OR > 1 implied a worse parameter correlated with elevated TUG1 expression. Heterogeneity assessment was conducted by a Chi-square-based *Q* statistical test and Higgins *I*-squared statistic. When the inconsistency index (*I*^2^) ≥ 50% or *p* < 0.10, this indicated that there was substantial heterogeneity among the studies, and a random effects model was applied. When *I*^2^ < 50% or *p* < 0.10, a fixed effects pattern was used. Sensitivity analysis was performed to assess the robustness of the overall results. Begg's test was conducted to determine the potential publication bias, with *p* < 0.05 indicating a clear publication bias. *p* values less than 0.05 were considered statistically significant.

## 3. Results

### 3.1. Study Characteristics

**Figure 1:**
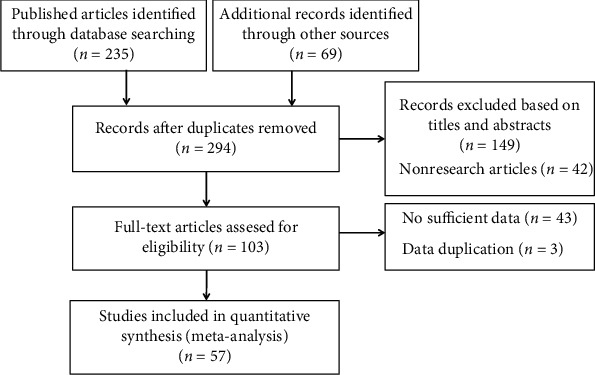
Flow diagram of the selection procedure for the studies.

According to the strategy depicted in Figure 1, a total of 57 eligible studies involving 8753 patients were included to assess the association of TUG1 with survival and clinicopathological characteristics [4, 6, 9, 10, 19–71]. Among them, Zhou et al. [40] contained 8 eligible cohorts with different types of tumors, and Gradia et al. [62] analyzed two subtypes of breast cancer. These cohorts were analyzed separately. The detailed characteristics of the included studies are summarized in Table 1, which shows that we included 8753 patients, and the studies were published from 2014 to 2021. Among the 57 studies (65 cohorts), 48 cohorts were conducted in China, 12 cohorts data were extracted from the Cancer Genome Atlas (TCGA), and the remaining three cohorts were performed in Egypt, Germany, and the Czech Republic. Thirty-seven of the included cohorts enrolled less than 100 patients and 24 cohorts recruited more than 100 patients. The incorporated cancer types included glioblastoma (GBM) [10, 42, 63], nasopharyngeal carcinoma (NPC) [49], oral squamous cell carcinoma (OSCC) [58], esophageal squamous cell carcinoma (ESCC) [6, 21, 29, 67], breast cancer (BC) [28, 40, 62], small cell lung cancer (SCLC) [53], non-small-cell lung cancer (NSCLC) [9, 19, 25, 69], hepatocellular carcinoma (HCC) [40], gastric cancer (GC) [23, 24, 40, 41, 65, 66], cholangiocarcinoma (CCA) [60, 61], pancreatic carcinoma (PC) [35, 50, 57], colorectal cancer(CRC) [4, 39, 40, 46, 51, 52], renal cell carcinoma(RCC) [54], urothelial carcinoma(UC) [56], bladder cancer(BLC) [31, 36, 40, 68, 70], prostate cancer (PCa) [22, 26, 37, 38], cervical cancer (CC) [27, 59], endometrial carcinoma (EC) [32], ovarian cancer (OC) [20, 30, 44], osteosarcoma (OSA) [40, 45, 55, 71], acute myeloid leukemia (AML) [33, 43, 47, 48], non-Hodgkin's lymphoma (NHL) [64], and melanoma [34, 40]. RNA sequencing and qRT-PCR methods were used to determine TUG1 expression level, and the median value was applied as a cut-off value in most studies. As to the prognostic analysis, 61 cohorts evaluated the prognostic impact of TUG1 on OS, and 12 cohorts reported the impact of TUG1 on RFS, DFS, RFS, and EFS.

### 3.2. Correlation of TUG1 Expression with Survival

**Figure 2:**
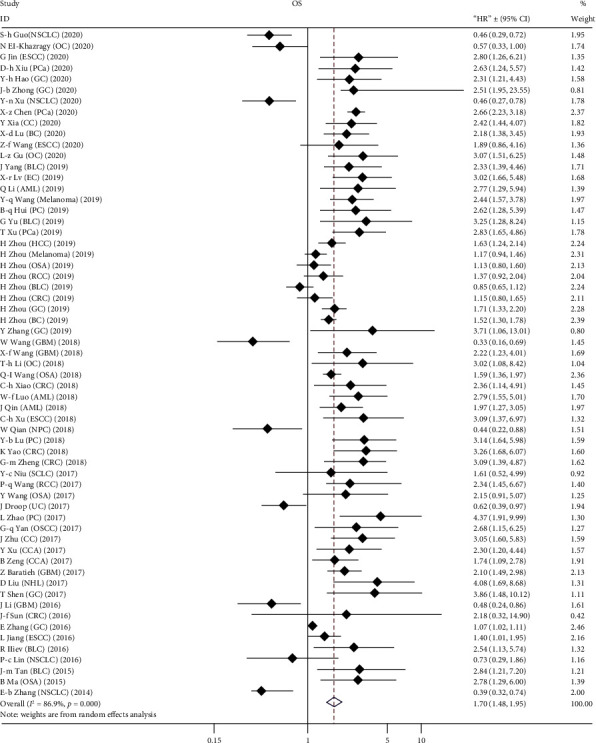
Forest plot for the association between high TUG1 expression and OS of patients with different tumor types.

**Figure 3:**
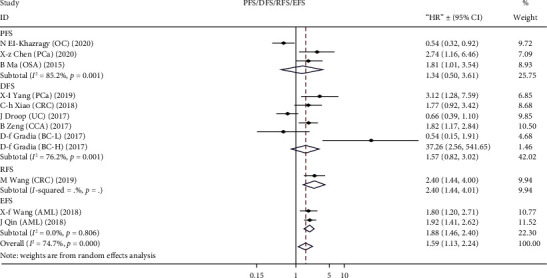
Forest plot for the relationship between high TUG1 expression and PFS/DFS/RFS/EFS of patients with different tumor types.

A total of 8405 patients were enrolled to assess the association between TUG1 level and OS. A random effect model was employed due to clear heterogeneity (*I*^2^ = 86.9%, *p* < 0.001). A significant correlation was found between high TUG1 expression and unfavorable OS, and the pooled HR was 1.70 (95% CI: 1.48-1.95, *p* < 0.001) (Figure 2). Twelve cohorts were included to investigate the relationship between TUG1 expression and PFS, DFS, RFS, and EFS. The results showed that TUG1 expression was not significantly correlated with PFS (*p* = 0.648) or DFS (*p* = 0.437), but a tendency toward worse RFS (*p* = 0.001) or EFS (*p* < 0.001) was revealed in patients with high level of TUG1 expression, although the number of included studies was extremely small (Figure 3).

### 3.3. Subgroup Analyses of the Correlation between High TUG1 Expression and OS in Cancer

**Figure 4:**
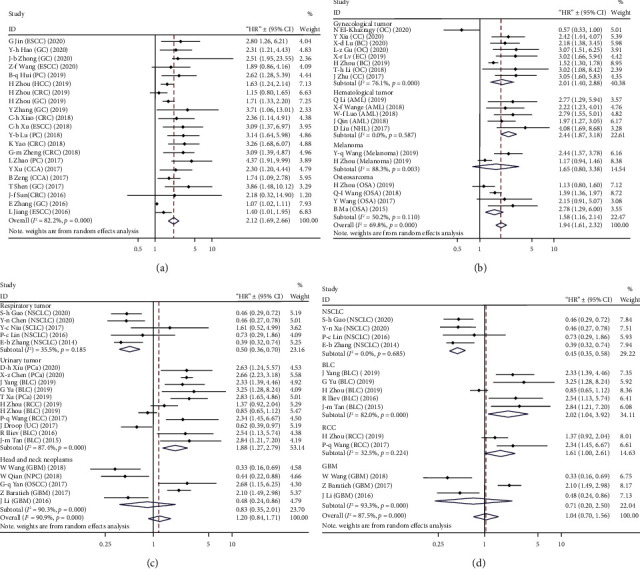
Stratified analyses for the association between high TUG1 expression with OS by cancer type. (a) Gastrointestinal cancer. (b) Gynecologic tumor, hematological tumor, melanoma, or osteosarcoma. (c) Respiratory tumor, urinary tumor, or head and neck neoplasm. (d) Non-small-cell lung cancer, bladder cancer, renal cell carcinoma, or glioblastoma.

To address the heterogeneity among OS datasets, we performed subgroup analyses according to patients' origin, cancer type, sample size, and detection method (Table 2). The results revealed a marked association between high expression of TUG1 and unfavorable OS in patients from China (HR = 1.93, 95% CI: 1.59-2.30, *p* < 0.001) and patients who were not from China (HR = 1.27, 95% CI: 1.05-1.54, *p* = 0.013). Likewise, TUG1 overexpression predicted a worse outcome no matter in the subgroup containing patients more than 100 (HR = 1.31, 95% CI: 1.08-1.60, *p* < 0.001) and in the subgroup with less than 100 patients (HR = 2.12, 95% CI: 1.72-2.63, *p* = 0.007). When grouped according to TUG1 detection method, the pooled HRs for the qRT-PCR subgroup and RNA sequencing subgroup were 1.88 (95% CI: 1.57-2.25, *p* < 0.001) and 1.29 (95% CI: 1.07-1.57, *p* = 0.009), respectively. When sorted by cancer type, TUG1 expression significantly predicted unfavorable OS in gastrointestinal cancer (HR = 2.12, 95% CI: 1.69-2.67, *p* < 0.001), urinary tumors (HR = 1.89, 95% CI: 1.27-2.79, *p* = 0.002), gynecologic tumors (HR = 2.01, 95% CI: 1.40-2.89, *p* < 0.001), hematological tumors (HR = 2.44, 95% CI: 1.87-3.18, *p* < 0.001), and osteosarcoma (HR = 1.58, 95% CI: 1.16-2.14, *p* = 0.003). Meanwhile, a high TUG1 level predicted favorable OS in respiratory tumors (HR = 0.50, 95% CI: 0.36-0.70, *p* < 0.001). TUG1 expression had no significant prognostic value in head and neck neoplasms and melanoma (Figures 4(a)–4(c)). Still, further stratified analysis indicated that elevated TUG1 exhibited a favorable prognostic value for NSCLC (HR = 0.45, 95% CI: 0.35-0.58, *p* < 0.001) and an unfavorable prognostic value for RCC (HR = 1.61, 95% CI: 1.00-2.61, *p* = 0.046). However, the merged HR indicated no significant relationship between TUG1 expression and OS in BLC (*p* = 0.441) and GBM (*p* = 1.135) (Figure 4(d)). Significant heterogeneity existed in all subgroups except for the hematological tumor subgroup (Table 2).

### 3.4. Impact of High TUG1 Expression on Clinicopathological Parameters

Clinicopathological analyses were conducted according to common parameters, such as patients' age, gender, tumor grade, tumor stage, lymph node metastasis, and distant metastasis. 1332 patients in fifteen cohorts were collected to assess the relationship between TUG1 expression and tumor differentiation. A significant connection was found between high TUG1 expression and low tumor differentiation in cancer patients, and the pooled OR was 1.99 (95% CI: 1.10-3.60, *p* = 0.023) with statistical heterogeneity (*I*^2^ = 80.7%, *p* < 0.001). Twenty cohorts with 1828 patients showed an association between TUG1 overexpression and tumoral TNM stage. The pooled OR was 2.82 (95% CI: 1.84-4.33, *p* < 0.001) with significant heterogeneity (*I*^2^ = 72.2%, *p* < 0.001), demonstrating that patients with up-regulated TUG1 expression are more likely to develop higher tumor stage. Subsequently, we investigated the prognostic value of TUG1 with lymph node metastasis and distant metastasis. The results indicated that patients with evaluated TUG1 expression progress to lymph node metastasis and distant metastasis by comparing the incidence of lymph node metastasis (HR = 2.96, 95% CI: 2.23-3.92, *p* < 0.001) and distant metastasis (HR = 3.56, 95% CI: 1.97-6.41, *p* < 0.001) between the high and low TUG1 expression groups. However, no significant correlation was detected for age or gender, and the pooled ORs are shown in Table 3.

### 3.5. Sensitivity Analysis

**Figure 5:**
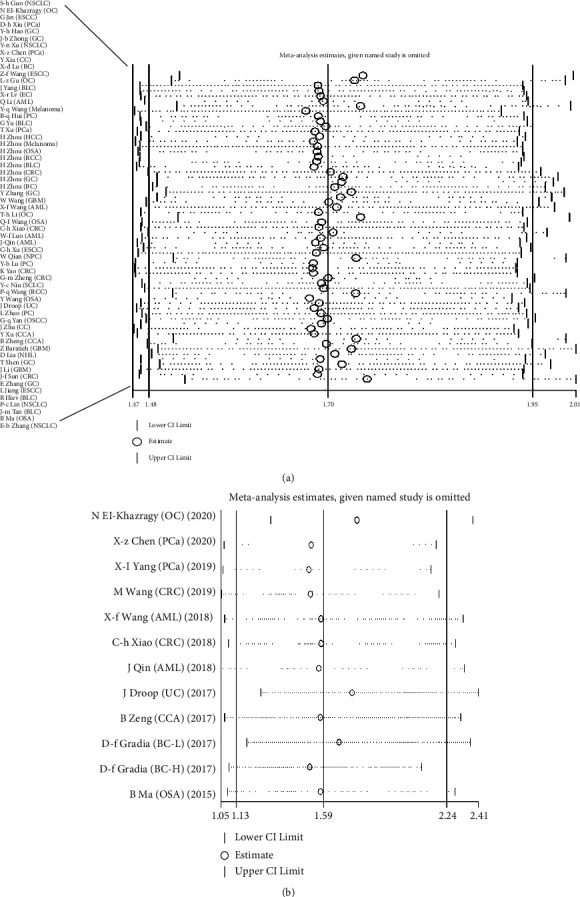
Sensitivity analysis for the meta-analysis of OS (a) and DFS/RFS/EFS (b) in all patients.

Sensitivity analysis was conducted to evaluate the robustness of the overall outcome. The pooled HRs were recalculated after excluding each single cohort successively, and the results indicated that the HR of high TUG1 expression on OS ranged from 1.67 (95% CI: 1.47-1.91) to 1.75 (95% CI: 1.53-2.01) (Figure 5(a)), and the HR of high TUG1 expression on PFS/DFS/RFS/EFS ranged from 1.51 (95% CI: 1.06-2.16) to 1.77 (95% CI: 1.31-2.38) (Figure 5(b)), suggesting that a positive association between high TUG1 level and the prognosis of cancer patients existed no matter which study was removed.

### 3.6. Publication Bias

**Figure 6:**
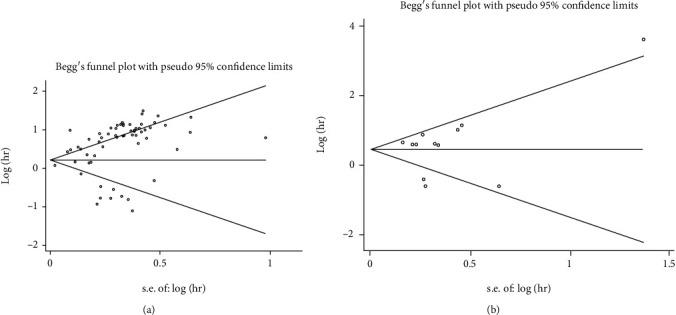
Funnel plot analysis of potential publication bias for OS (a) and DFS/RFS/EFS (b).

The potential for publication bias was assessed by funnel plots and Begg's test. The shape of the funnel plots for OS or PFS/DFS/RFS/EFS were asymmetric (Figure 6), but the *p* value of the Begg's test for OS (Pr > ∣*z* | = 0.61) and DFS/RFS/EFS (Pr > ∣*z* | = 0.95) indicated that there was no severe publication bias in our present meta-analysis.

## 4. Discussion

lncRNAs participate in regulating tumoral biological processes by competitively interacting with certain microRNAs, altering the expression of key component proteins in the gene regulatory system [72, 73]. To date, numerous studies have confirmed that mutation or misregulation of lncRNAs may promote tumorigenesis and metastasis and show that they are novel biomarkers and therapeutic targets for cancer [3, 74, 75].

TUG1, a 7.1 kb lncRNA, was first identified as a transcript upregulated in response to taurine treatment, which affects mouse retinal development [76]. Increasing numbers of studies have revealed that TUG1 is related to tumors. In most malignancies, TUG1 has been reported to be overexpressed and be involved in regulating of multiple processes in tumor progression, invasion and angiogenesis [77]. It has been confirmed by immunoprecipitation that TUG1 may recruit and bind to polycomb repressive complex 2 (PRC2) to regulate gene expression involved in tumorigenesis and tumor development [78, 79]. Additionally, TUG1 can also exert its oncogenic role via sponging tumoral suppressor microRNAs or modulating cancer-related signaling pathways like Wnt, MAPK, or Notch1 [80–82]. However, TUG1 was found to be downregulated and acted as a tumor suppressing gene in some types of breast cancer, NSCLC, and glioma [8–10]. For example, TUG1 can promote tumor cell apoptosis and inhibit the growth of glioma by activating caspase 3- and caspase 9-mediated proapoptosis, inhibiting bcl-2 mediated antiapoptosis [10, 83]. Thus, the prognostic-predictive value of TUG1 in cancer is still uncertain and needs further evaluation. The expression level of TUG1 in tumors and the correlation of TUG1 with patients' survival and clinicopathological characteristics have been previously assessed. In this study, we collected specific data of TUG1 involvement in tumor progression and survival of patients with different types of tumors, and we analyzed and summarized whether TUG1 is suitable as a prognostic marker for these tumors.

Although multiple meta-analyses have suggested that TUG1 could be used as a tumor-related prognostic marker, most studies were conducted before 2017 [12–18]. The prognostic value of TUG1 in some particular types of tumors is still controversial, and its clinical application is relatively limited. With the development of tumoral genome sequencing technology, more data on TUG1 have been published over the past few years. In this study, a total of 57 articles (65 cohorts) were included to comprehensively analyze the role of TUG1 in 22 types of tumors from across the body, and the results provide more information for TUG1 as a tumor prognostic biomarker to be applied in clinical prognostic risk analysis. Additionally, almost all the incorporated research data were from China in the previous analyses. Our research included multiple TCGA cohorts to increase the sample size and the diversity of the data, making the research results more convincing.

In our study, the results suggest that high TUG1 expression is significantly associated with worse OS in patients with malignant tumors, which is consistent with the conclusions drawn from previous studies [13, 14, 16–18]. In addition, the correlation between TUG1 expression and PFS/DFS/RFS/EFS in patients with tumors was analyzed for the first time. No significant association between TUG1 expression and PFS/DFS was found. Meanwhile, since only one included paper reported RFS and two articles reported EFS, the prognostic value of TUG1 on PFS/EFS is uncertain, even though we observed a significant *p* value by survival analysis.

In the subgroup analysis, we found that the high expression of TUG1 was related to poor OS of patients with gastrointestinal cancers (ESCC, GC, CRC, PC, HCC, and CCA), gynecological tumors (BC, OC, CC, and EC), hematological tumors (AML and NHL), urinary tumors (RCC, BLC, UC, and PCa), and OSA. This result was not reported in previous analyses, which may be due to the limited number of eligible studies for each tumor type. In urinary tumors, only one study on UC showed that a high expression of TUG1 was significantly related to better prognosis (HR = 0.62, 95% CI: 0.39-0.97, *p* = 0.012). BLC included five eligible studies. One study enrolling 406 patients from the TCGA database suggested that patients with high expression of TUG1 tended to have better prognosis, but no statistical difference was found [40]. Another 4 studies included 256 bladder cancer patients, 3 studies from China and 1 from the Czech Republic, with the results suggesting that patients with higher TUG1 expression have a lower survival rate. Although the overall analysis of urinary system tumors confirmed that the high expression of TUG1 has a prognostic value for patients, it is still necessary to expand the sample size to evaluate whether TUG1 plays a role in UC that can be distinguished from other urinary tumors, and the prognostic value of TUG1 in BLC also needs to be further verified. In respiratory tumors, 4 NSCLC studies indicated that patients with upregulated TUG1 levels have better prognosis (HR = 0.46, 95% CI: 0.27-0.80, *p* = 0.061), which was opposite to other tumor types. While only one SCLC article showed that patients with upregulated TUG1 expression tend to have worse prognosis, no statistical significance was found. The difference in the role of TUG1 in NSCLS and SCLC must be clarified by expanding the included studies in the future, and the specific lung tissue and pathological type may determine the prognostic role of TUG1 in lung cancer. In head and neck neoplasms and malignant melanoma, the expression of TUG1 was not significantly correlated with the survival of tumor patients. Head and neck neoplasm analysis included 3 GBM, 1 NPC, and 1 OSCC study. Of the 3 GBM articles, 2 were from China, indicating that patients with high TUG1 level have better prognosis. One TCGA data analysis was contrary to the previous two studies, suggesting that patients with low TUG1 expression have a longer survival time. Two melanoma studies displayed an association between upregulated TUG1 and poor prognosis, with no statistical significance. All these tumors need to be further studied to clarify whether TUG1 has prognostic value. The subgroup analysis based on patients' origin, sample size, and TUG1 detection method suggested that the prognostic value of TUG1 was not affected by these factors.

Furthermore, we analyzed the clinicopathological parameters related to TUG1. Different from previous analyses, the high expression of TUG1 was positively associated with tumor TNM stage, tumor differentiation, lymph node metastasis, and distant metastasis, which further confirmed the meaningful prognostic value of TUG1 in various tumors.

There are some limitations in the current study, and it should be interpreted with caution. First, the survival analysis data were not provided directly in some studies and needed to be extracted from Kaplan-Meier curves. However, some Kaplan-Meier curves were relatively difficult to extract due to the low pixel count [69] or the large sample size [40, 63], and the data obtained may contain errors. For example, the *p* value displayed in Zhou et al.' work suggested that no statistical difference was found between TUG1 expression and the survival of patients in gastric cancer or breast cancer, whereas a statistical difference appeared in these two tumors through repeated extraction and calculation [40]. Thus, we utilized the data acquired by actual extraction for statistical analysis. Second, the prognostic role of TUG1 in head and neck neoplasms and malignant melanoma has not been confirmed. This may be due to the limitation of the study samples, and more sample analysis is needed in the future. Third, there was statistical heterogeneity among the studies included in this research. This may be due to differences in tumor types, the number of cases, the patient sources, the detection methods, and the cut-off values of TUG1. Of note, the difference in TUG1 cut-off values and units may influence the application of TUG1 in the clinic.

## 5. Conclusions

Despite the limitations described above, our meta-analysis still showed that elevated TUG1 is significantly related to favorable prognosis of respiratory tumors and poor prognosis of gastrointestinal cancers, gynecological tumors, hematological tumors, urinary tumors, and osteosarcoma. No definite conclusion has been reached for head and neck neoplasms and malignant melanoma, and further analysis with a larger sample size is needed. Furthermore, the high expression of TUG1 is significantly associated with late tumor stage, poor differentiation, more lymph node metastases, and distant metastasis of tumors.

## Figures and Tables

**Table 1:** Characteristics summary of the 57 eligible studies in this meta-analysis.

Study	Year	Origin of population	Cancer type	Sample number	lncRNA TUG1 high/low	Stage	Detection method	Study endpoints	Hazard ratios	NOS
S-h Guo	2020	China	NSCLC	132	52/80	I-IV	qRT-PCR	OS	K-M	6
N El-Khazragy	2020	Egypt	OC	100	50/50	I-IV	qRT-PCR	OS/PFS	K-M	6
G Jin	2020	China	ESCC	50	27/23	I-IV	qRT-PCR	OS	K-M	6
D-h Xiu	2020	China	PCa	50	25/25	I-IV	qRT-PCR	OS	K-M	7
Y-h Hao	2020	China	GC	110	80/30	I-IV	qRT-PCR	OS	K-M	8
J-b Zhong	2020	China	GC	83	49/34	I-IV	qRT-PCR	OS	HR	7
Y-n Xu	2020	China	NSCLC	79	45/34	I-IV	qRT-PCR	OS	K-M	8
X-z Chen	2020	China	PCa	54	16/38	I-IV	qRT-PCR	OS/PFS	HR/K-M	8
Y Xia	2020	China	CC	137	69/68	I-II	qRT-PCR	OS	HR/K-M	8
X-d Lu	2020	China	BC	90	52/38	I-III	qRT-PCR	OS	HR/K-M	8
Z-f Wang	2020	TCGA	ESCC	86	72/14	NA	RNA-seq	OS	K-M	6
L-z Gu	2020	China	OC	41	21/20	II-IV	qRT-PCR	OS	K-M	6
J Yang	2019	China	BLC	68	38/30	II-IV	qRT-PCR	OS	HR/K-M	8
X-r Lv	2019	China	EC	58	37/21	I-IV	qRT-PCR	OS	HR/K-M	8
Q Li	2019	China	AML	36	18/18	NA	qRT-PCR	OS	K-M	7
Y-q Wang	2019	China	Melanoma	40	NA	NA	qRT-PCR	OS	HR/K-M	5
B-q Hui	2019	China	PC	42	21/21	I-IV	qRT-PCR	OS	K-M	8
G Yu	2019	China	BLC	87	44/43	NA	qRT-PCR	OS	K-M	6
X-l Yang	2019	China	PCa	46	23/23	NA	qRT-PCR	DFS	K-M	6
T Xu	2019	China	PCa	70	35/35	I-IV	qRT-PCR	OS	K-M	7
M Wang	2019	China	CRC	124	62/62	II-III	qRT-PCR	RFS	K-M	8
H Zhou	2019	TCGA	HCC	371	91/274	NA	RNA-seq	OS	K-M	5
H Zhou	2019	TCGA	Melanoma	459	115/344	NA	RNA-seq	OS	K-M	5
H Zhou	2019	TCGA	OSA	259	65/194	NA	RNA-seq	OS	K-M	5
H Zhou	2019	TCGA	RCC	287	73/214	NA	RNA-seq	OS	K-M	5
H Zhou	2019	TCGA	BLC	406	102/304	NA	RNA-seq	OS	K-M	5
H Zhou	2019	TCGA	CRC	279	69/210	NA	RNA-seq	OS	K-M	5
H Zhou	2019	TCGA	GC	392	93/299	NA	RNA-seq	OS	K-M	5
H Zhou	2019	TCGA	BC (triple negative)	1081	273/808	NA	RNA-seq	OS	K-M	5
Y Zhang	2019	China	GC	85	48/37	I-IV	qRT-PCR	OS	K-M	7
W Wang	2018	China	GBM	51	NA	NA	RNA-seq	OS	HR	5
X-f Wang	2018	China	AML	186	93/93	NA	qRT-PCR	OS/EFS	HR/K-M	7
T-h Li	2018	China	OC	96	NA	I-IV	qRT-PCR	OS	HR/K-M	7
Q-l Wang	2018	China	OSA	94	47/47	IIA/IIB-III	qRT-PCR	OS	HR/K-M	8
C-h Xiao	2018	China	CRC	90	45/45	I-IV	qRT-PCR	OS/DFS	K-M	7
W-f Luo	2018	China	AML	73	NA	NA	qRT-PCR	OS	HR/K-M	6
J Qin	2018	China	AML	236	NA	NA	qRT-PCR	OS/EFS	HR/K-M	6
C-h Xu	2018	China	ESCC	42	21/21	NA	qRT-PCR	OS	K-M	7
W Qian	2018	China	NPC	48	28/20	I-IV	qRT-PCR	OS	K-M	8
Y-b Lu	2018	China	PC	72	50/22	I-IV	qRT-PCR	OS	K-M	8
K Yao	2018	China	CRC	185	129/56	I-IV	qRT-PCR	OS	HR/K-M	8
G-m Zheng	2018	China	CRC	90	51/39	I-IV	qRT-PCR	OS	HR/K-M	8
Y-c Niu	2017	China	SCLC	33	16/17	NA	qRT-PCR	OS	K-M	7
P-q Wang	2017	China	RCC (ccRCC)	203	100/103	I-IV	qRT-PCR	OS	HR/K-M	8
Y Wang	2017	China	OSA	44	30/14	I-IIA/IIB-III	qRT-PCR	OS	K-M	8
J Droop	2017	Germany	UC	106	NA	I-IV	qRT-PCR	OS/DFS	HR/K-M	8
L Zhao	2017	China	PC	34	18/16	I-IV	qRT-PCR	OS	K-M	8
G-q Yan	2017	China	OSCC	46	24/24	I-IV	qRT-PCR	OS	HR/K-M	8
J Zhu	2017	China	CC	59	30/29	I-IIA/IIB-IIIA	qRT-PCR	OS	K-M	8
Y Xu	2017	China	CCA	51	30/29	I-IV	qRT-PCR	OS	K-M	8
B Zeng	2017	China	CCA	102	NA	I-IV	qRT-PCR	OS/DFS	HR/K-M	7
D-f Gradia	2017	TCGA	BC (luminal B)	122	92/30	I-IV	RNA-seq	DFS	HR/K-M	5
D-f Gradia	2017	TCGA	BC (HER2-enriched)	56	14/42	I-IV	RNA-seq	DFS	HR/K-M	5
Z Baratieh	2017	TCGA	GBM	260	130/130	NA	RNA-seq	OS	K-M	5
D Liu	2017	China	NHL	108	61/47	I-IV	qRT-PCR	OS	HR/K-M	8
T Shen	2017	China	GC	48	35/13	I-IV	qRT-PCR	OS	K-M	8
J Li	2016	China	GBM	120	60/60	I-IV	qRT-PCR	OS	K-M	8
J-f Sun	2016	China	CRC	120	72/48	I-IV	qRT-PCR	OS	K-M	8
E Zhang	2016	China	GC	100	50/50	I-IV	qRT-PCR	OS	HR/K-M	8
L Jiang	2016	China	ESCC	218	109/109	I-IV	qRT-PCR	OS	HR/K-M	8
R Iliev	2016	Czech Republic	BLC	47	26/21	II-IV	qRT-PCR	OS	HR/K-M	7
P-c Lin	2016	China	NSCLC	89	31/58	I-IV	qRT-PCR	OS	K-M	8
J-m Tan	2015	China	BLC	54	27/27	I-IV	qRT-PCR	OS	K-M	7
B Ma	2015	China	OSA	76	41/35	I-III	qRT-PCR	OS/PFS	HR/K-M	8
E-b Zhang	2014	China	NSCLC	192	96/96	I-IV	qRT-PCR	OS	K-M	6

Abbreviations: AML: acute myeloid leukemia; BC: breast cancer; BLC: bladder cancer; CC: cervical cancer; CCA: cholangiocarcinoma; CRC: colorectal cancer; EC: endometrial carcinoma; ESCC: esophageal squamous cell carcinoma; GBM: glioblastoma; GC: gastric cancer; HCC: hepatocellular carcinoma; NHL: non-Hodgkin's lymphoma; NPC: nasopharyngeal carcinoma; NSCLC: non-small-cell lung cancer; OC: ovarian cancer; OSA: osteosarcoma; OSCC: oral squamous cell carcinoma; PC: pancreatic carcinoma; PCa: prostate cancer; RCC: renal cell carcinoma; SCLC: small cell lung cancer; UC: urothelial carcinoma.

**Table 2:** Subgroup analysis of the pooled HRs of OS with overexpressed lncRNA TUG1 in patients with cancer.

Stratified analysis	Number of cohorts	Number of patients	Pooled HR (95% CI)	*p* value	Heterogeneity	
					*I* ^2^ (%)	*p* value
Origin						
China	48	4272	1.93 (1.59-2.30)	<0.001	88.3	<0.001
Non-China	13	4133	1.27 (1.05-1.54)	0.013	76.6	<0.001
Cancer type						
Head and neck neoplasms	5	525	0.83 (0.35-2.01)	0.687	90.3	<0.001
Respiratory tumor	5	525	0.50 (0.36-0.70)	<0.001	35.5	0.185
Gastrointestinal cancer	21	2650	2.12 (1.69-2.67)	<0.001	82.2	<0.001
Urinary tumor	11	1432	1.89 (1.27-2.79)	0.002	87.4	<0.001
Gynecologic tumor	8	1662	2.01 (1.40-2.89)	<0.001	76.5	<0.001
Hematological tumor	5	639	2.44 (1.87-3.18)	<0.001	0	0.587
Osteosarcoma	4	473	1.58 (1.16-2.14)	0.003	50.2	0.110
Melanoma	2	499	1.65 (0.80-3.38)	0.174	88.3	0.003
Number of patients						
<100	37	2256	2.12 (1.72-2.63)	0.007	88.3	<0.001
≥100	24	6149	1.31 (1.08-1.60)	<0.001	84.1	<0.001
Detection method						
qRT-PCR	50	4644	1.88 (1.57-2.25)	<0.001	88.0	<0.001
RNA-seq	11	4109	1.29 (1.07-1.57)	0.009	76.7	<0.001

**Table 3:** Correlation between lncRNA TUG1 and clinicopathological characteristics of tumors.

Clinicopathologial features	Number of cohorts	Number of patients	Pooled OR (95%CI)	*p* value	Heterogeneity	
					*I* ^2^ (%)	*p* value
Gender (male vs. female)	31	1869	1.03 (0.84-1.25)	0.803	0	0.872
Age (<60 vs. ≥60)	15	1161	0.93 (0.73-1.18)	0.537	0	0.799
Tumor grade (low vs. high+moderate)	15	1332	1.99 (1.10-3.60)	0.023	80.7	<0.001
Tumor stage (III+IV vs. I+II)	20	1828	2.82 (1.84-4.33)	<0.001	72.2	<0.001
Lymph node metastasis (+ vs. -)	20	1835	2.96 (2.23-3.92)	<0.001	39.6	0.036
Distant metastasis (+ vs. -)	10	851	3.56 (1.97-6.41)	<0.001	57.1	0.013

## Data Availability

All data generated or analyzed during this study are included in this article.

## References

[1] Kong Y., Lu Z., Liu P. (2019). Long noncoding RNA: genomics and relevance to physiology. *Comprehensive Physiology*.

[2] Zhang X., Hong R., Chen W., Xu M., Wang L. (2019). The role of long noncoding RNA in major human disease. *Bioorganic Chemistry*.

[3] Bhan A., Soleimani M., Mandal S. S. (2017). Long noncoding RNA and cancer: a new paradigm. *Cancer research*.

[4] Sun J., Ding C., Yang Z. (2016). The long non-coding RNA TUG1 indicates a poor prognosis for colorectal cancer and promotes metastasis by affecting epithelial-mesenchymal transition. *Journal of Translational Medicine*.

[5] Guo P., Zhang G., Meng J., He Q., Li Z., Guan Y. (2018). Upregulation of long noncoding RNA TUG1 promotes bladder cancer cell proliferation, migration, and invasion by inhibiting miR-29c. *Oncology Research*.

[6] Xu C., Guo Y., Liu H., Chen G., Yan Y., Liu T. (2018). TUG1 confers cisplatin resistance in esophageal squamous cell carcinoma by epigenetically suppressing PDCD4 expression via EZH2. *Cell & Bioscience*.

[7] Li Y., Zhang T., Zhang Y., Zhao X., Wang W. (2018). Targeting the FOXM1-regulated long noncoding RNA TUG1 in osteosarcoma. *Cancer Science*.

[8] Fan S., Yang Z., Ke Z. (2017). Downregulation of the long non-coding RNA TUG1 is associated with cell proliferation, migration, and invasion in breast cancer. *Biomedicine & pharmacotherapy*.

[9] Zhang E. B., Yin D. D., Sun M. (2014). P53-regulated long non-coding RNA TUG1 affects cell proliferation in human non-small cell lung cancer, partly through epigenetically regulating HOXB7 expression. *Cell death & disease*.

[10] Li J., Zhang M., An G., Ma Q. (2015). LncRNA TUG1 acts as a tumor suppressor in human glioma by promoting cell apoptosis. *Experimental Biology and Medicine*.

[11] Li B., Shen S., Zhang W., Qi T., Hu Q., Cheng Y. (2018). Long non-coding RNA TUG1 as a potential novel biomarker for predicting the clinical outcome of cancer patients: a meta-analysis. *Clinical Laboratory*.

[12] Li N., Shi K., Kang X., Li W. (2017). Prognostic value of long non-coding RNA TUG1 in various tumors. *Oncotarget*.

[13] Ma P. J., Guan Q. K., Meng L., Qin N., Zhao J., Jin B. Z. (2017). Long non-coding RNA TUG1 as a potential prognostic biomarker in human cancers: a meta-analysis. *Oncotarget*.

[14] Wang X., Chen X., Zhang D. (2017). Prognostic and clinicopathological role of long non-coding RNA taurine upregulated 1 in various human malignancies: a systemic review and meta-analysis. *Tumour biology*.

[15] Yu X. H., Guo W., Zhang J. (2017). Long non-codingRNA (lncRNA) TUG1 and the prognosis of cancer: a meta-analysis. *Cellular and Molecular Biology (Noisy-le-Grand, France)*.

[16] Liu J., Lin J., Li Y., Zhang Y., Chen X. (2017). Prognostic role of lncRNA TUG1 for cancer outcome: evidence from 840 cancer patients. *Oncotarget*.

[17] Zhong Y., Chen Z., Guo S. (2017). TUG1, SPRY4-IT1, and HULC as valuable prognostic biomarkers of survival in cancer: a PRISMA-compliant meta-analysis. *Medicine*.

[18] Zhou Y., Lu Y., Li R., Yan N., Li X., Dai T. (2017). Prognostic role of long non-coding RNA TUG1 expression in various cancers: a meta-analysis. *Oncotarget*.

[19] Guo S., Yin Y., Wang Z. (2020). lncRNA TUG1 expression in NSCLC and its clinical significance. *Clinical Laboratory*.

[20] El-Khazragy N., Mohammed H. F., Yassin M. (2020). Tissue-based long non-coding RNAs " *PVT1*, *TUG1* and *MEG3* " signature predicts Cisplatin resistance in ovarian Cancer. *Genomics*.

[21] Jin G., Yang Y., Tuo G., Wang W., Zhu Z. (2020). LncRNA TUG1 promotes tumor growth and metastasis of esophageal squamous cell carcinoma by regulating XBP1 via competitively binding to miR-498. *Neoplasma*.

[22] Xiu D., Liu L., Cheng M., Sun X., Ma X. (2020). Knockdown of lncRNA TUG1 enhances radiosensitivity of prostate cancer via the TUG1/miR-139-5p/SMC1A axis. *Oncotargets and Therapy*.

[23] Hao Y. H., Cai Y. Q., Lu Y. L. (2020). Relationship of taurine up-regulated gene 1 and miRNA-145 in gastric cancer. *Chinese Archives of General Surgery (Electronic Edition)*.

[24] Zhong J. B., Wang Q., Wei J. C. (2020). Study on the relationship between the expression of long non-coding RNA ZDHHC8P1, MEG3, TUG1 and the clinicopathological characteristics and prognosis in gastric cancer. *Progress in Modern Biomedicine*.

[25] Xu Y. N., Yang X., Tao L. Q., Zhu J. F. (2020). Expressions and significance of lncRNA TUG1 and miR-132 in non-small cell lung cancer. *Jiangsu Medical Journal*.

[26] Chen X. Z., Meng Q. Z., Li P., Liu Z. Z., Shi F., Dong X. Q. (2020). The expression of long non-coding RNA TUG1 in prostate cancer tissues and its relationship with prognosis. *Journal of Modern Oncology*.

[27] Xia Y., Jin Z. J. (2020). Evaluation of long non-coding RNA taurine upregulated gene 1 expression level in diagnosis and prognosis of stage I-II cervical cancer. *Journal of Fujian Medical University*.

[28] Lu X. D. (2020). Expression of long non-coding RNA taurine upregulated gene 1 and miR-132 in breast cancer andtheir relationship with prognosis. *Chinese Archives of General Surgery (Electronic Edition)*.

[29] Wang Z., Liu J., Wang R., Wang Q., Liang R., Tang J. (2020). Long non-coding RNA taurine upregulated gene 1 (TUG1) downregulation constrains cell proliferation and invasion through regulating cell division cycle 42 (CDC42) expression via miR-498 in esophageal squamous cell carcinoma cells. *Medical science monitor*.

[30] Gu L., Li Q., Liu H., Lu X., Zhu M. (2020). Long noncoding RNA TUG1 promotes autophagy-associated paclitaxel resistance by sponging miR-29b-3p in ovarian cancer cells. *Oncotargets and Therapy*.

[31] Yang J., Cheng F. (2019). Expression of long non-coding RNA TUG1 in urothelial carcinoma of the bladder and its value in predicting clinical prognosis. *Journal of Clinical Urology*.

[32] Lv X. R., Yue H. (2019). The expression and clinical significance of lncRNA PVT1,H19 and TUG1 in patients with endometrial carcinoma. *Journal of Modern Oncology*.

[33] Li Q., Song W., Wang J. (2019). TUG1 confers adriamycin resistance in acute myeloid leukemia by epigenetically suppressing miR-34a expression via EZH2. *Biomedicine & pharmacotherapy*.

[34] Wang Y., Liu G., Ren L., Wang K., Liu A. (2019). Long non-coding RNA TUG1 recruits miR‑29c‑3p from its target gene RGS1 to promote proliferation and metastasis of melanoma cells. *International journal of oncology*.

[35] Hui B., Xu Y., Zhao B. (2019). Overexpressed long noncoding RNA TUG1 affects the cell cycle, proliferation, and apoptosis of pancreatic cancer partly through suppressing RND3 and MT2A. *Oncotargets and Therapy*.

[36] Yu G., Zhou H., Yao W., Meng L., Lang B. (2019). lncRNA TUG1 promotes cisplatin resistance by regulating CCND2 via epigenetically silencing miR-194-5p in bladder cancer. *Molecular therapy Nucleic acids*.

[37] Yang X. L., Wei C., Zhang Y. B., Guo H. Q. (2019). Long noncoding RNA TUG1 promotes progression via upregulating DGCR8 in prostate cancer. *European review for medical and pharmacological sciences*.

[38] Xu T., Liu C. L., Li T., Zhang Y. H., Zhao Y. H. (2019). LncRNA TUG1 aggravates the progression of prostate cancer and predicts the poor prognosis. *European review for medical and pharmacological sciences*.

[39] Wang M., Hu H., Wang Y. (2019). Long non-coding RNA TUG1 mediates 5-fluorouracil resistance by acting as a ceRNA of miR-197-3p in colorectal cancer. *Journal of Cancer*.

[40] Zhou H., Sun L., Wan F. (2019). Molecular mechanisms of TUG1 in the proliferation, apoptosis, migration and invasion of cancer cells. *Oncology letters*.

[41] Zhang Y., Liang C. J., Guo J. S. (2019). The expression of lncRNA-TUG1 in gastric cancer tissues and its significance. *Journal of Chinese Oncology*.

[42] Wang W., Zhao Z., Yang F. (2018). An immune-related lncRNA signature for patients with anaplastic gliomas. *Journal of Neuro-Oncology*.

[43] Wang X., Zhang L., Zhao F. (2018). Long non-coding RNA taurine-upregulated gene 1 correlates with poor prognosis, induces cell proliferation, and represses cell apoptosis via targeting aurora kinase A in adult acute myeloid leukemia. *Annals of Hematology*.

[44] Li T. H., Zhang J. J., Liu S. X., Chen Y. (2018). Long non-coding RNA taurine-upregulated gene 1 predicts unfavorable prognosis, promotes cells proliferation, and inhibits cells apoptosis in epithelial ovarian cancer. *Medicine*.

[45] Wang Q., Chen Q. (2018). Role of taurine upregulated gene 1 as a predictor of poor outcome in osteosarcoma. *Journal of cancer research and therapeutics*.

[46] Xiao C. H., Yu H. Z., Guo C. Y. (2018). Long non-coding RNA TUG1 promotes the proliferation of colorectal cancer cells through regulating Wnt/*β*-catenin pathway. *Oncology letters*.

[47] Luo W., Yu H., Zou X., Ni X., Wei J. (2018). Long non-coding RNA taurine-upregulated gene 1 correlates with unfavorable prognosis in patients with refractory or relapsed acute myeloid leukemia treated by purine analogue based chemotherapy regimens. *Cancer biomarkers.*.

[48] Qin J., Bao H., Li H. (2018). Correlation of long non-coding RNA taurine-upregulated gene 1 with disease conditions and prognosis, as well as its effect on cell activities in acute myeloid leukemia. *Cancer biomarkers.*.

[49] Qian W., Ren Z., Lu X. (2019). Knockdown of long non-coding RNA TUG1 suppresses nasopharyngeal carcinoma progression by inhibiting epithelial-mesenchymal transition (EMT) via the promotion of miR-384. *Biochemical and biophysical research communications*.

[50] Lu Y., Tang L., Zhang Z. (2018). Long noncoding RNA TUG1/miR-29c axis affects cell proliferation, invasion, and migration in human pancreatic cancer. *Disease Markers*.

[51] Yao K., Zhu G. H., Shan Y. Z. (2018). Expressions of long noncoding RNA TUG1 and UCA1 in colon cancer tissue and their clinical significance. *Chinese Journal of General Surgery*.

[52] Zheng G. M., Feng Q. L. (2018). Expression and clinical significance of serum lncRNA Tug1 expression in colorectal cancer patients. *Chinese Journal of Current Advances in General Surgery*.

[53] Niu Y., Ma F., Huang W. (2017). Long non-coding RNA TUG1 is involved in cell growth and chemoresistance of small cell lung cancer by regulating LIMK2b via EZH2. *Molecular cancer*.

[54] Wang P. Q., Wu Y. X., Zhong X. D., Liu B., Qiao G. (2017). Prognostic significance of overexpressed long non-coding RNA TUG1 in patients with clear cell renal cell carcinoma. *European review for medical and pharmacological sciences*.

[55] Wang Y., Yang T., Zhang Z. (2017). Long non-coding RNA TUG1 promotes migration and invasion by acting as a ceRNA of miR-335-5p in osteosarcoma cells. *Cancer Science*.

[56] Droop J., Szarvas T., Schulz W. A. (2017). Diagnostic and prognostic value of long noncoding RNAs as biomarkers in urothelial carcinoma. *PLoS One*.

[57] Zhao L., Sun H., Kong H., Chen Z., Chen B., Zhou M. (2017). The Lncrna-TUG1/EZH2 Axis promotes pancreatic cancer cell proliferation, migration and EMT phenotype formation through sponging Mir-382. *Cellular Physiology and Biochemistry*.

[58] Yan G., Wang X., Yang M., Lu L., Zhou Q. (2017). Long non-coding RNA TUG1 promotes progression of oral squamous cell carcinoma through upregulating FMNL2 by sponging miR-219. *American Journal of Cancer Research*.

[59] Zhu J., Shi H., Liu H., Wang X., Li F. (2017). Long non-coding RNA TUG1 promotes cervical cancer progression by regulating the miR-138-5p-SIRT1 axis. *Oncotarget*.

[60] Xu Y., Leng K., Li Z. (2017). The prognostic potential and carcinogenesis of long non-coding RNA TUG1 in human cholangiocarcinoma. *Oncotarget*.

[61] Zeng B., Ye H., Chen J. (2017). LncRNA TUG1 sponges miR-145 to promote cancer progression and regulate glutamine metabolism via Sirt3/GDH axis. *Oncotarget*.

[62] Gradia D. F., Mathias C., Coutinho R., Cavalli I. J., Ribeiro E., de Oliveira J. C. (2017). Long non-coding RNA TUG1 expression is associated with different subtypes in human breast cancer. *Noncoding RNA*.

[63] Baratieh Z., Khalaj Z., Honardoost M. A. (2017). Aberrant expression of PlncRNA-1 and TUG1: potential biomarkers for gastric cancer diagnosis and clinically monitoring cancer progression. *Biomarkers in Medicine*.

[64] Liu D., Li X. M. (2017). Expression of taurine upregulated gene 1 in the patients with DLBCL and its relationship with prognosis of the patients. *Chinese Journal of Cancer Biotherapy*.

[65] Shen T., Si J. L., Cui J. Y., Qi Y. Q., Lv M. (2017). Expression of long non-coding RNA TUG1 and its effect on prognosis of patients with gastric cancer. *Chinese Journal of Gastroenterology*.

[66] Zhang E., He X., Yin D. (2016). Increased expression of long noncoding RNA TUG1 predicts a poor prognosis of gastric cancer and regulates cell proliferation by epigenetically silencing of p57. *Cell death & disease*.

[67] Jiang L., Wang W., Li G. (2016). High TUG1 expression is associated with chemotherapy resistance and poor prognosis in esophageal squamous cell carcinoma. *Cancer Chemotherapy and Pharmacology*.

[68] Iliev R., Kleinova R., Juracek J. (2016). Overexpression of long non-coding RNA TUG1 predicts poor prognosis and promotes cancer cell proliferation and migration in high-grade muscle-invasive bladder cancer. *Tumour biology*.

[69] Lin P.-C., Huang H.-D., Chang C.-C. (2016). Long noncoding RNA TUG1 is downregulated in non-small cell lung cancer and can regulate CELF1 on binding to PRC2. *BMC Cancer*.

[70] Tan J., Qiu K., Li M., Liang Y. (2015). Double-negative feedback loop between long non-coding RNA TUG1 and miR-145 promotes epithelial to mesenchymal transition and radioresistance in human bladder cancer cells. *FEBS Letters*.

[71] Ma B., Li M., Zhang L. (2016). Upregulation of long non-coding RNA TUG1 correlates with poor prognosis and disease status in osteosarcoma. *Tumour biology.*.

[72] Zhang Y., Tao Y., Liao Q. (2018). Long noncoding RNA: a crosslink in biological regulatory network. *Briefings in Bioinformatics*.

[73] Chan J. J., Tay Y. (2018). Noncoding RNA:RNA regulatory networks in cancer. *International journal of molecular sciences*.

[74] Wang J., Zhang X., Chen W., Hu X., Li J., Liu C. (2019). Regulatory roles of long noncoding RNAs implicated in cancer hallmarks. *International journal of cancer*.

[75] Tang Y., Cheung B. B., Atmadibrata B. (2017). The regulatory role of long noncoding RNAs in cancer. *Cancer letters*.

[76] Young T. L., Matsuda T., Cepko C. L. (2005). The noncoding RNA _taurine upregulated gene 1_ is required for differentiation of the murine retina. *Current Biology*.

[77] Ghaforui-Fard S., Vafaee R., Taheri M. (2019). Taurine-upregulated gene 1: a functional long noncoding RNA in tumorigenesis. *Journal of cellular physiology*.

[78] Khalil A. M., Guttman M., Huarte M. (2009). Many human large intergenic noncoding RNAs associate with chromatin-modifying complexes and affect gene expression. *Proceedings of the National Academy of Sciences of the United States of America*.

[79] van Kruijsbergen I., Hontelez S., Veenstra G. J. C. (2015). Recruiting polycomb to chromatin. *The International Journal of Biochemistry & Cell Biology*.

[80] Liu S., Liu Y., Lu Q., Zhou X., Chen L., Liang W. (2018). The lncRNA TUG1 promotes epithelial ovarian cancer cell proliferation and invasion via the WNT/beta-catenin pathway. *Oncotargets and Therapy*.

[81] Tian L., Zhao Z. F., Xie L., Zhu J. P. (2019). Taurine up-regulated 1 accelerates tumorigenesis of colon cancer by regulating miR-26a-5p/MMP14/p38 MAPK/Hsp27 axis _in vitro_ and _in vivo_. *Life Sciences*.

[82] Xu K., Zhang L. (2020). Inhibition of TUG1/miRNA-299-3p axis represses pancreatic cancer malignant progression via suppression of the Notch1 pathway. *Digestive diseases and sciences*.

[83] Li J., An G., Zhang M., Ma Q. (2016). Long non-coding RNA TUG1 acts as a miR-26a sponge in human glioma cells. *Biochemical and biophysical research communication.*.

